# Identification of a core set of rhizobial infection genes using data from single cell-types

**DOI:** 10.3389/fpls.2015.00575

**Published:** 2015-07-28

**Authors:** Da-Song Chen, Cheng-Wu Liu, Sonali Roy, Donna Cousins, Nicola Stacey, Jeremy D. Murray

**Affiliations:** ^1^State Key Laboratory of Agricultural Microbiology, College of Life Science and Technology, Huazhong Agricultural University, WuhanChina; ^2^John Innes Centre, Department of Cell and Developmental Biology, NorfolkUK

**Keywords:** infection threads, methoxychalcone, medicarpin, CCAAT-box, infection zone, nod genes, Nod factors, nodulation

## Abstract

Genome-wide expression studies on nodulation have varied in their scale from entire root systems to dissected nodules or root sections containing nodule primordia (NP). More recently efforts have focused on developing methods for isolation of root hairs from infected plants and the application of laser-capture microdissection technology to nodules. Here we analyze two published data sets to identify a core set of infection genes that are expressed in the nodule and in root hairs during infection. Among the genes identified were those encoding phenylpropanoid biosynthesis enzymes including *Chalcone-O-Methyltransferase* which is required for the production of the potent Nod gene inducer 4′,4-dihydroxy-2-methoxychalcone. A promoter-GUS analysis in transgenic hairy roots for two genes encoding *Chalcone-O-Methyltransferase* isoforms revealed their expression in rhizobially infected root hairs and the nodule infection zone but not in the nitrogen fixation zone. We also describe a group of *Rhizobially Induced Peroxidases* whose expression overlaps with the production of superoxide in rhizobially infected root hairs and in nodules and roots. Finally, we identify a cohort of co-regulated transcription factors as candidate regulators of these processes.

## Introduction

Most legumes are able to interact with soil bacteria called rhizobia to form special root structures called nodules within which the bacteria are able to fix atmospheric nitrogen, a process which is fueled by host photosynthate. In *Medicago truncatula*, as with many legumes, the rhizobia infects the host root by colonizing special intracellular tubular invaginations called infection threads that form first in root hairs and then later in mature nodules. This process begins with rhizobial attachment near the tip of growing root hairs which then curl and entrap a rhizobial microcolony in an infection pocket, also called an infection focus. The infection thread then extends as a tubular structure from this pocket through the length of the root hair, becoming colonized by rhizobia as it grows (Fournier et al., 2008). The process of infection thread formation is then repeated in the underlying cell layers allowing the rhizobia access to the inner root layers which have divided to form the NP. Root hair infection involves the upregulation of 100s of genes that regulate several different processes including host-symbiont signaling and diverse developmental processes including cell growth and engagement of the cell cycle (Libault et al., 2010; Breakspear et al., 2014). While maturing the nodule forms several developmental zones: an apical meristem (Zone I), an infection zone containing cells that form infection threads (ZII), a nitrogen fixing zone comprised of giant cells filled with endocytosed nitrogen-fixing rhizobia (ZIII), an interzone (IZ), and a senescent zone, where no nitrogen fixation takes place (ZIV). The process of infection, from the initial entry of the rhizobia into the infection threads up until their release into symbiosomes, requires constant communication between the host and symbiont. At the heart of the dialog is the production of bacterial signaling compounds called Nod factors. Nod factors are produced in response to plant flavonoids, phenylpropanoid compounds which are secreted by the roots into the rhizosphere (Peters et al., 1986; Redmond et al., 1986; Maxwell et al., 1989; Kape et al., 1992; Phillips et al., 1994; Zuanazzi et al., 1998; Subramanian et al., 2007). The secreted flavonoids induce the expression of rhizobial nod genes required for the production of Nod factors through activation of the transcription factor NodD (Mulligan and Long, 1985; Rossen et al., 1985). The flavonoids produced by a given host are often specific to certain symbionts, only activating Nod genes in nodulation-competent rhizobia (Györgypal et al., 1988). In *M. truncatula*, several flavonoids capable of inducing Nod gene in its symbiont *Sinorhizobium meliloti* have been identified and one of the most potent is 4,4′-dihydroxy 2′-methoxychalcone (Kapulnik et al., 1987; Maxwell et al., 1989; Orgambide et al., 1994). The enzyme Chalcone *O*-Methyltransferase (ChOMT) in the closely related *M. sativa* is required for production of this compound from isoliquiritigenin (4,2′,4′-trihydroxychalcone; Maxwell et al., 1992). Our recent study has shown that the *M. truncatula* ortholog, *ChOMT1*, and three other close homologs (*ChOMT2*, *ChOMT3*, and *ChOMT4*) were induced during infection of root hairs (Breakspear et al., 2014). Transcripts for two of these isoforms were also detected in the infection zone (Zone II) of mature nodules (Roux et al., 2014), but a spatio-temporal analysis of the expression of these genes during early infection is lacking.

One of the first physiological events occurring during nodulation is the generation of ROS (Cook et al., 1995; Ramu et al., 2002), which is coincident with enhanced flavonoid biosynthesis (van Brussel et al., 1990; Mathesius et al., 1998; Hassan and Mathesius, 2012). Earlier studies on the production and breakdown of ROS by rhizobia suggest that ROS levels must be maintained between certain limits for the symbiosis to be successful (Santos et al., 2000; Jamet et al., 2003, 2007). The expression of *Rhizobial Induced Peroxidase 1* (*RIP1*) was shown to increase in response to rhizobial infection or Nod factors and was found to be correlated with the production of ROS during rhizobial infection (Cook et al., 1995; Ramu et al., 2002). Recently, transcriptomic analysis of root hairs revealed that *RIP1* belongs to a family of 10 *RIPs* that are similarly strongly induced upon inoculation with rhizobia or Nod factors, further suggesting an important role for the modulation of ROS during nodulation (Breakspear et al., 2014).

In this study we compared published data from studies using single-cell types, i.e., microarray data for root hairs collected from seedlings inoculated with rhizobia (Breakspear et al., 2014) and RNAseq data for laser-capture microdissected nodules (Roux et al., 2014) to identify a core set of genes associated with infection. We then investigated the expression of two *ChOMT* genes and the generation of superoxides during the different stages of nodulation in infected root hair and nodule cells. Finally, we identified transcription factors induced during rhizobial infection of root hairs and preferentially expressed in the nodule infection zone as potential regulators of ROS and phenylpropanoid production.

## Materials and Methods

### Analysis of Transcriptomics Data

The first data set used for our analysis was from Roux et al. (2014). This study generated RNAseq data by laser-capture microdissection of *M. truncatula* cv. Jemalong A17 nodules harvested 15 days post inoculation with *S. meliloti* 2011. This was designated as data set A. A second data set was from a microarray analysis of root hairs of harvested from *M. truncatula* cv. Jemalong A17 seedlings 1, 3, and 5 days post inoculation with *S. meliloti* 1021 or treated with Nod factor (24 h post treatment; Breakspear et al., 2014), designated data set B. Roux et al. (2014) assigned all plant and bacterial genes that were differentially expressed between zones into 13 hierarchical clusters based on their expression across the sampled nodule zones. To compare the two data sets we identified all genes in dataset B that could be assigned to the above-mentioned clusters and compared their frequencies to that of all genes assigned to clusters in dataset A using a chi-squared test with the online GraphPad QuickCalc software http://graphpad.com/quickcalcs/chisquared1.cfm.

### Plant Growth Conditions

Composite plants and seedlings were grown in controlled environment chambers with 16 h day length with a light intensity of 90–130 μmol m^-2^ s^-1^ and constant temperature of 20°C. For rhizobial inoculation a 24-h culture of *S. meliloti* 1021 rhizobia was spun down and resuspended in buffered nodulation medium to an optical density of 0.02 at 600 nm, and 3 mL of the culture was used to inoculate each of the growing *M. truncatula* plants.

### Promoter-GUS Analysis

For the promoter-GUS analyses, the promoter regions of *ChOMT2* (Medtr3g021440; from -23 to -1803 bp) and *ChOMT3* (Medtr7g011900; from -23 to -1885 bp) were amplified by PCR using Phusion High Fidelity DNA polymerase (NEB). The fragments were then cloned into pDONR207 and after sequence confirmation were transferred to the destination vector pKGWFS7 upstream of the GUS open reading frame using the GATEWAY cloning system (Life Technologies) as per the manufacturer’s recommended protocol. The vector was then transformed into *M. truncatula* (A17) using *Agrobacterium rhizogenes*-mediated hairy root transformation as described previously (Breakspear et al., 2014). The composite plants with transgenic roots were then transferred to a soil mixture of Terragreen (Oil-Dri UK) and silver sand 1:1 mixture, inoculated with *S. meliloti* 1021, and watered as needed with distilled water. Nodulated roots were then stained for GUS activity at 1, 2, and 3 weeks post inoculation, as previously described (Breakspear et al., 2014).

### Nitroblue tetrazolium (NBT) Staining

For nitroblue tetrazolium (NBT) staining, *M. truncatula* seedlings were grown in Terragreen (Oil-Dri UK) and silver sand mixture (1:1) for 7 days and then inoculated with *S. meliloti* 1021. At 14 days post inoculation the nodulated roots were cut off and stained in 0.1% NBT water solution (Promega UK) for 40 min in the dark at room temperature. Imbedding and sectioning of plant tissues was carried out as previously described (Guan et al., 2013).

## Results

Infection threads form in both root hairs and in cells of nodule ZII. To identify genes common to infection thread formation in these two cell types we compared genes that were induced either by rhizobia or by purified Nod factors in root hairs with the genes expressed in different nodule zones (Roux et al., 2014). To do this we used the RNAseq analysis described by Roux et al. (2014) which assigned genes to 13 clusters based on their expression across tissues sampled from each nodule zone using laser-capture microdissection. The advantage of this approach is that genes that are only expressed in the nodule but not in the root hair or vice versa are not considered. For example, the large family genes encoding for nodule-specific cysteine-rich (NCR) peptides, which are highly expressed in the nodule but are not expressed in root hairs, are thereby excluded. A total of 768 genes were identified that met the clustering criteria and were induced by at least one treatment in root hairs. When these genes were assigned to their clusters a disproportionate number of the rhizobially induced genes in the WT and *sickle* (*skl*) mutant belonged to clusters 2–5, which are genes that are primarily expressed in the nodule meristem (ZI) and distal ZII with some expression in proximal ZII (**Table 1**). Nod factor-induced genes displayed an overlapping pattern, with clusters 1–4 being over-represented, cluster one containing genes with the majority of their expression in the meristem (ZI) with some expression in distal ZII (**Table 1**). For each treatment and the combined treatments the observed frequencies in the different clusters were significantly different than the expected frequencies (Chi-squared tests, all *p*-values <0.0001). This analysis suggests that a core set of genes underlies infection processes in both tissue types.

**Table 1:** Comparison of the observed number of genes induced by rhizobial inoculation in root hairs belonging to different clusters of nodule expression (Roux et al., 2014).

	RHIZ + WT		RHIZ + *skl*		Nod factors + WT		All treatments	
Cluster^1^	Obs	Exp	Obs	Exp	Obs	Exp	Obs	Exp
							


1	13	16.5	25	31.9	14	11.9	34	38.9
	


2	75	31.0	108	59.8	57	22.4	131	72.9
	


3	41	17.1	69	32.9	31	12.3	79	40.1
	


4	78	33.0	143	63.5	34	23.8	168	77.4
				


5	55	52.6	142	101.4	28	38.0	166	123.6
6	6	24.1	12	46.4	14	17.4	24	56.6
7	4	9.9	8	19.1	7	7.2	12	23.3
8	5	8.2	6	15.8	4	5.9	9	19.2
9	3	25.5	4	49.1	5	18.4	9	59.8
10	3	10.2	5	19.7	0	7.4	6	24.0
11	17	37.8	39	72.9	16	27.3	47	88.8
12	13	21.9	23	42.1	12	15.8	30	51.4
13	14	39.1	46	75.4	14	28.2	53	91.9

#genes^2^	*327*		*630*		*236*		*768*	
%^3^	76.1%		73.3%		57.6%		70.8%	


*^1^clusters from Roux et al. (2014).*

*^2^number of genes induced by rhizobia or Nod factor (Breakspear et al., 2014).*

*^3^ percentage of genes in overrepresented clusters (shaded cells).*

**FIGURE 1:**
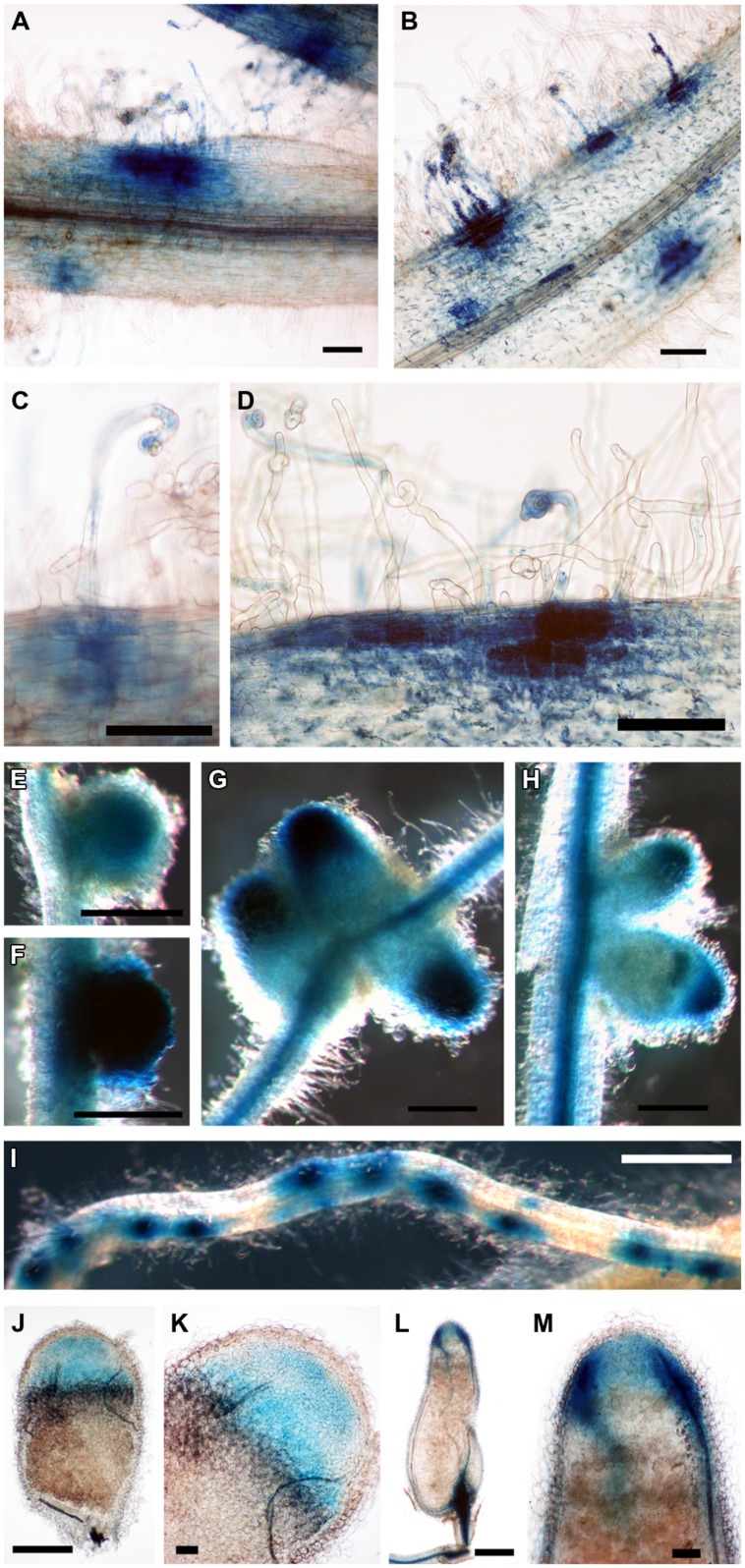
**Expression of *Chalcone O-Methyltransferase* (*ChOMT2*) and *ChOMT3* during nodulation of *Medicago truncatula*.** **(A,C,E,G,J,K)** *pCHOMT2:GUS* transgenic roots 7–21 days post inoculation with *Sinorhizobium meliloti* 1021. **(B,D,F,H,I,L,M)** *pCHOMT3:GUS* transgenic roots 7–21 days post inoculation with *S. meliloti* 1021. Bars are 100 μm **(A,B,C,D,K,M)**, 500 μm **(E,F,G,H,J,L)**, and 1000 μm **(I)**. **(J,K,L,M)** are free-hand sections.

We then considered the genes that were induced by rhizobial infection and were found in the over-represented clusters 2–5 (Supplementary Table S1). Amongst these genes were several members of the isoflavonoid biosynthesis pathway analyzed in Breakspear et al. (2014) including *Chalcone Reductase (CHR)* which is required for the production of isoliquiritigenin and *ChOMT* which converts isoliquiritigenin to the even more potent nod gene inducer methoxychalcone. Four *ChOMT* genes are induced in root hairs after rhizobial infection (Breakspear et al., 2014), but their expression patterns during nodulation have not been investigated in detail. We investigated the expression of two of these genes, *ChOMT2* and *ChOMT3,* during the early stages of infection and nodule formation using promoter-GUS analysis in *A. rhizogenes* transformed roots. We found that both genes were expressed in root hairs undergoing infection (**Figures 1A–D**) and throughout the NP (**Figures 1E,F**). As the nodule matured the expression of both genes was restricted to the apex (**Figures 1G,H**), and in root tips (data not shown). The expression of both genes was typically observed at numerous infection sites along the root (**Figures 1A,B,D,I**). Hand sectioning of the nodules revealed expression of *ChOMT2* in the nodule meristem, the infection zone, with some expression in the IZ, closely matching the published LCM data (**Figures 1J,K**; Roux et al., 2014). *ChOMT3* expression was also found in the nodule meristem and but was absent from the IZ and proximal infection zone (**Figures 1L,M**), again closely reflecting the data of Roux et al. (2014). Expression of both genes was absent in the nitrogen fixation zone. *ChOMT3* expression was also strong in the apical part of the nodule vasculature, including the nodule vascular meristems (**Figures 1L,M**).

**FIGURE 2:**
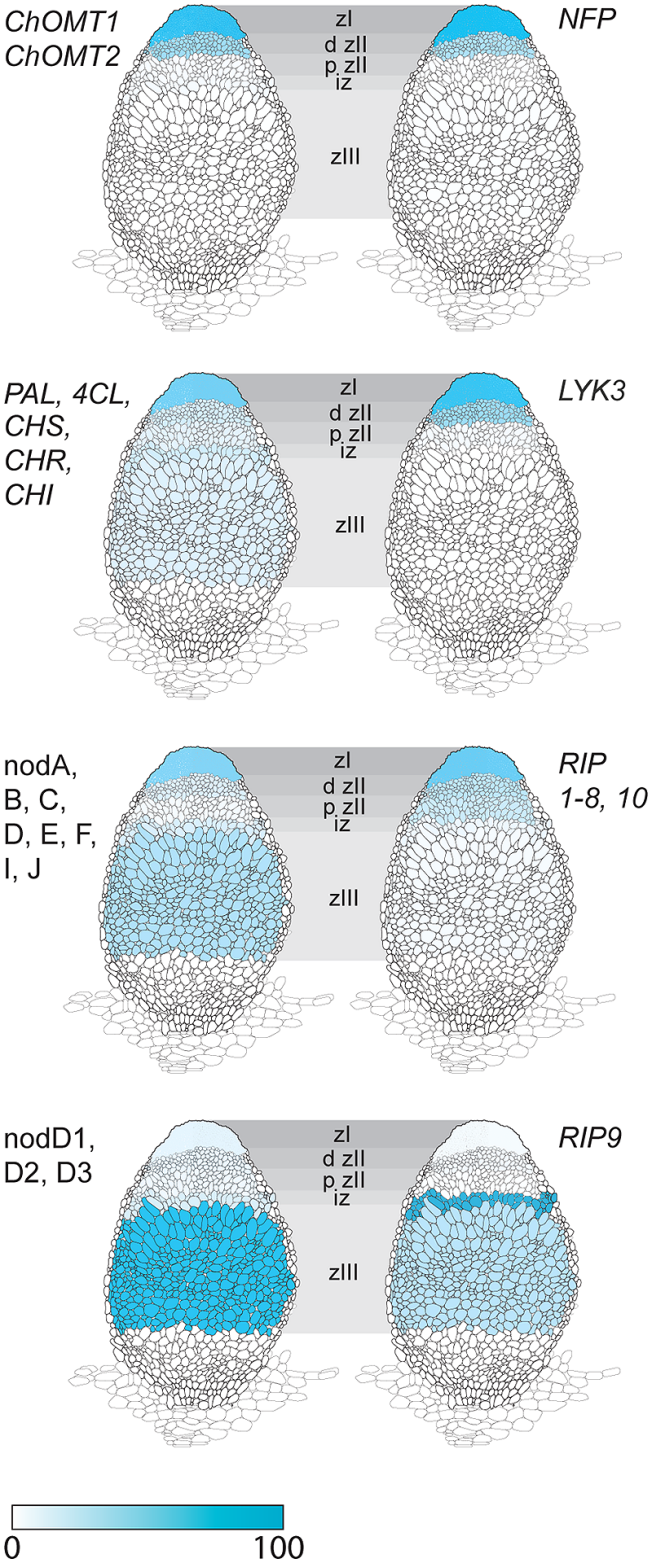
**Expression in different nodule zones of a family of *Rhizobial Induced Peroxidases (RIPs)* and genes involved in the induction of Nod factor biosynthesis by flavonoids.** Genes for isoliquiritigenin biosynthesis: *ChOMT1*, *ChOMT2*; genes *Phenylalanine Ammonia Lyase* (*PAL*), *4-Coumarate:coA Ligase* (*4CL*), *Chalcone Synthase* (*CHS*), *Chalcone Reductase* (*CHR*), *Chalcone Isomerase* (*CHI*); Rhizobial nod genes for Nod factor biosynthesis and transport: nodA, B, C, D, E, F, I, J; *S. meliloti* nod (nodD1, D2, D3) genes for flavonoid-dependent activation of Nod factor biosynthesis; Nod factor receptor genes *Nod Factor Perception* (*NFP*) and *LysM receptor-like kinase* (*LYK3*); *RIPs 1–10.* Data, adapted from Roux et al. (2014), is represented as percent of total normalized transcripts for each gene or group of genes, see Supplementary Table S2 for details.

The expression of *ChOMT2* and *ChOMT3* corresponded well with the other genes encoding enzymes for isoliquiritigenin production which had an average of 65% of their total expression in ZI and ZII (**Figure 2**, Supplementary Table S1). However, some transcripts of these genes (∼10% of the total) were also detected in each of the remaining zones (IZ, ZIII). The expression of the rhizobial nod genes required for the biosynthesis and secretion of Nod factors which are known to be induced by flavonoids also had strong expression in ZI and proximal ZII (on average 55% of the total reads), however, as noted by Roux et al. (2014), they also showed expression (∼25% of total reads) in the nitrogen fixation zone (ZIII; **Figure 2**). Based on our results it seems unlikely that methoxychalcone is responsible for the induction of nod gene expression in ZIII. Instead this expression could be induced by isoliquiritigenin for which the required genes are expressed in ZIII (**Figure 2**). In contrast to the Nod factor biosynthesis and transport genes, the three *nodD* genes were mostly expressed in ZIII, with only one (nodD3) having some expression in the nodule apex (ZI; **Figure 2**).

### Rhizobial Induced Peroxidases

**FIGURE 3:**
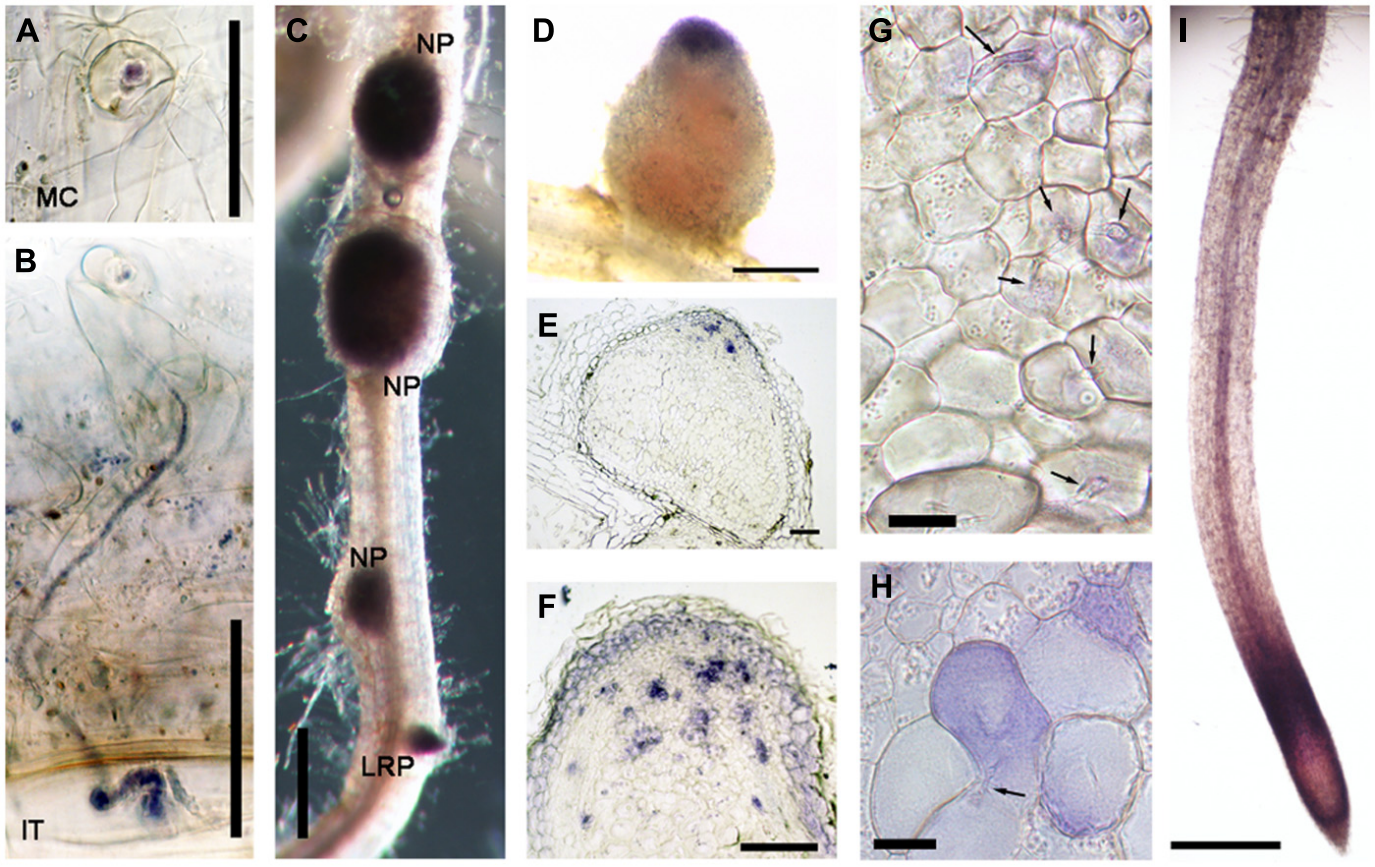
**Nitroblue tetrazolium (NBT) staining of *M. truncatula* roots nodulated by *S. meliloti* 1021. (A)** A microcolony within an infection focus. **(B)** An infection thread in a root hair. **(C)** Nodule primordia (NP) and a lateral root primordium (LRP). **(D)** An intact nodule **(E,F,G,H)** nodule sections, 10 μm thick **(I)** the primary root tip. In **(G)** and **(H)** arrows indicate infection threads. Plants harvested 14 dpi with *S. meliloti* 1021. Bars = 50 μm **(A,B)**, 100 μm **(E,F)**, and 500 μm **(C,D,I)**, 20 μm **(G,H)**.

Another gene class identified in the overlap between the inoculated root hair and nodule data sets was type III peroxidases, which are apoplastic/cell wall localized enzymes have a role in cell wall remodeling (Francoz et al., 2015). The genes encoding this family of enzymes have been shown to be inducible by rhizobia and Nod factors in root hairs and designated as *RIP1-10* (Breakspear et al., 2014). Most of the previously identified *RIPs* are also present in ZI and ZII (**Figure 2**, data from Roux et al., 2014). Unexpectedly one of them, *RIP9*, was more highly expressed than all the others combined (Supplementary Table S3) and had a dramatically different expression domain, being >90% expressed in the IZ and ZIII (**Figure 2**). Since secreted peroxidases are capable of generating reactive oxygen species, we monitored superoxide production at the different stages of nodulation using NBT staining (Doke, 1983). The stain was seen in microcolonies and in infection threads at the early stages of nodulation (**Figures 3A,B**). The nascent NP were also strongly stained (**Figure 3C**) and as nodules matured, the staining was mainly found in the nodule apex (**Figure 3D**). Nodule sectioning revealed staining in a patchwork of individual cells located mostly in ZII and sometimes in ZIII (**Figures 3E,F**). Closer inspection revealed that the staining was present in cells containing infection threads in ZII (**Figure 3G**) and also in ZIII (**Figure 3H**). There was also staining in a thin layer of non-infected cells near the nodule apex (**Figure 3F**). In addition, staining was also strong in root tips of both mature primary roots and emerging lateral roots (**Figure 3G**).

### Transcription Factors

The transcription factors controlling the expression of genes for flavonoid biosynthesis and ROS production in the nodule are currently not known. A large number of transcription factors (**Table 2**) were shared between the genes in nodule clusters 2–5 and genes induced in root hairs of infected plants (**Table 3**). These included the well-studied *ERN1*, *ERN2,* and *NSP2* which are required for early infection events and nodule development (Oldroyd and Long, 2003; Kaló et al., 2005; Heckmann et al., 2006; Middleton et al., 2007; Cerri et al., 2012). In addition, four genes encoding CCAAT-box transcription factor subunits (*NF-YA1*, *NF-YA2*, *NF-YC2*, *NF-YB7*) were in this group. *NF-YA1* and *NF-YC2* are important for both rhizobial infection and nodule development (Combier et al., 2006, 2008; Zanetti et al., 2010; Soyano et al., 2013; Laporte et al., 2014), while *NF-YA2* is a close homolog of *NF-YA1*. This co-regulation across root hair and nodule-zones suggests that the encoded subunits may act as part of heterocomplex along with NF-YB7 to regulate infection. Notably the *nf-ya1* mutant forms nodules with either reduced or absent meristems that fail to release the bacteria from the infection thread into symbiosomes (Xiao et al., 2014). A summary of all data (Breakspear et al., 2014; Roux et al., 2014) for the entire family of *M. truncatula* CCAAT-box transcription factors is included in Supplementary Table S4.

**Table 2:** Transcription factors associated with rhizobial infection.

Gene	Gene model/Position^3^	Class	Cluster	^4^F1%	FIID%	FIIP%	IZ%	ZIII%
ZFP6 (At)	Chr7 31519725-31520145	zinc finger	2			000.0	00.5	00.0
MYB3R-1 (At)	Medtr3g110028	MYB	2		24.5	04.1	00.0	00.2
NSP2	Medtr3g072710	GRAS	2	57.5	31.7	09.9	00.7	00.1
WRKY40 (At)	Medtr2g105060	WRKY	2	47.4			07.9	00.0
ERN1	Medtr7g085810	AP2	2	55.8		07.9	00.1	00.0
BTF3 (At)	Medtr3g020660	BTF3b-like	4	41.7	26.9	18.7	07.2	05.5
	Medtr7g105790	ovate	4	46.6	25.4	22.0	05.1	00.9
WRKY23 (At)	Medtr7g109800	WRKY	4	46.5	36.0	08.2	03.1	6.2
ERF12 (At)	Medtr2g014300	ERF	4	45.7	34.7	13.3	03.2	03.1
ERF9 (At)	Medtr4g078710	ERF	4	38.6	36.6	13.6	05.4	5.7
MYB6 (At)	Medtr3g097450	MYB	4	30.4	36.0	13.8	13.7	06.2
NF-YA1^1^	Medtr1g056530	CCAAT-box	3	26.3	33.6	27.2	08.5	04.4
CRF4 (At)	Medtr3g090760	ERF	3	26.0	37.8	25.4	07.1	03.7
	Medtr6g092540	MYB-like	3	23.9	48.2		05.7	05.1
NF-YC2	Medtr7g113680	CCAAT-box	3	21.2	34.6	012.3	18.8	13.1
	Medtr8g101650	MYB-like	3	22.1	45.5	10.9	08.9	12.6
NF-YA2	Medtr7g106450	CCAAT-box	3	17.3	45.6	31.5	04.7	00.9
ERN2	Medtr6g029180	AP2	3	09.8	65.9	10.6	13.1	00.7
RRA3^2^	Medtr3g088630	RR	3	12.0	55.5	30.8	01.5	00.1
N20	Medtr7g086040	GRF-ZF	3	10.2	63.0	26.6	00.3	00.0
NF-YB7	Medtr8g091720	CCAAT-box	3	02.9	41.3	51.2	04.6	00.0


*Data from Roux et al. (2014).*

*Cells shading indicates relative strength of expression for a given gene across nodule zones.*

*^1^Previously HAP2.1.*

*^2^Previously MtARR8, see notes on family nomenclature (Liu et al., 2015).*

*^3^Chromosome position Mtv4.0.*

*^4^Percentage of normalized RNAseq reads across samples (Roux et al., 2014).*

The roles of the remaining transcription factors in nodulation have not been defined. Of note in this group are the nodule-specific GRF-Zinc finger gene N20, and the *M. truncatula* ortholog of *Arabidopsis thaliana* Zinc Finger Protein 6 (ZFP6). ZFP6 has been assigned a role in integration of gibberellic acid and cytokinin signaling (Zhou et al., 2013).

**Table 3:** Transcription factors associated with rhizobial infection.

			Fold change vs. control				
Gene name	Gene model/Position	*probeset*	1 dpi^1^	3 dpi	5 dpi	5 dpi *skl*	24 h NF^2^
ZFP6 (At)	Chr7 31519725-31520145	Mtr.32254.1.S1_at	02.0	01.5	06.4^∗^	09.0^∗^	01.6
MYB3R-1 (At)	Medtr3g110028	Mtr.36181.1.S1_at	01.0	01.1	02.1	02.6	02.1^∗^
NSP2	Medtr3g072710	Mtr.44789.1.S1_at	01.6^∗^	00.9	01.7	03.0^∗^	00.7^∗^
WRKY40 (At)	Medtr2g105060	Mtr.10896.1.S1_s_at	00.5	00.7	00.8	00.8	02.6^∗^
ERN1	Medtr7g085810	Mtr.7556.1.S1_at	01.5^∗^	01.2	03.9^∗^	08.9^∗^	02.5^∗^
BTF3 (At)	Medtr3g020660	Mtr.12254.1.S1_x_at	01.2	01.4	01.5^∗^	02.1^∗^	01.2
	Medtr7g105790	Mtr.25635.1.S1_at	03.4	03.3	04.3	22.9^∗^	01.1
WRKY23 (At)	Medtr7g109800	Mtr.25503.1.S1_at	01.3	01.6^∗^	01.5	02.4^∗^	00.9
ERF12 (At)	Medtr2g014300	Mtr.985.1.S1_at	08.7^∗^	15.8^∗^	13.7^∗^	19.4^∗^	05.4^∗^
ERF9 (At)	Medtr4g078710	Mtr.26158.1.S1_at	03.3^∗^	03.2^∗^	04.7^∗^	23.5^∗^	02.9^∗^
MYB6 (At)	Medtr3g097450	Mtr.1797.1.S1_at	01.2	01.2	02.1	03.3	02.4^∗^
NF-YA1	Medtr1g056530	Mtr.43750.1.S1_at	36.9^∗^	157.1^∗^	114.5^∗^	549.6^∗^	59.0^∗^
CRF4 (At)	Medtr3g090760	Mtr.41691.1.S1_at	02.6	02.8	05.3^∗^	08.5^∗^	01.5
	Medtr6g092540	Mtr.25945.1.S1_at	01.9^∗^	02.8	05.6^∗^	09.6^∗^	10.5^∗^
NF-YC2	Medtr7g113680	Mtr.48660.1.S1_at	01.7^∗^	02.1	02.0^∗^	04.6^∗^	02.0^∗^
	Medtr8g101650	Mtr.9513.1.S1_at	13.1	11.2^∗^	09.8^∗^	10.3^∗^	11.6^∗^
NF-YA2	Medtr7g106450	Mtr.1584.1.S1_at	01.7^∗^	02.8	02.9^∗^	05.6^∗^	03.6^∗^
ERN2	Medtr6g029180	Mtr.43947.1.S1_at	01.2	00.8	01.8	02.6^∗^	01.9^∗^
RRA3	Medtr3g088630	Mtr.31738.1.S1_at	02.3	07.4	12.1^∗^	42.6^∗^	01.0
N20	Medtr7g086040	Mtr.14503.1.S1_at	01.1	03.9	09.3^∗^	15.0^∗^	01.6
NF-YB7	Medtr8g091720	Mtr.4282.1.S1_at	10.2^∗^	03.2	03.0	02.0	05.1^∗^


*Data from Breakspear et al. (2014).*

*^1^dpi, days post inoculation with *Sinorhizobium meliloti* 1021.*

*^2^NF, 24 h post treatment with 10 nM Nod factors.*

*^∗^Significantly different compared to control *p* < 0.05, also shaded light red (see Breakspear et al., 2014 for details).*

## Discussion

### Root Hair Infection-Genes are Expressed in Nodule Zone II

Current molecular genetic studies of nodulation rely heavily on gene expression data to provide insight into symbiotic processes. Candidate genes identified in this manner can then be followed up using reverse genetic studies such as the *Tnt1* or *LORE1* transposon mutant collections available in *M. truncatula* or *Lotus japonicus*, respectively (Tadege et al., 2008; Cheng et al., 2011; Fukai et al., 2012; Urbański et al., 2012). Recently laser-capture has been used to target the specific regions of indeterminate nodules (Roux et al., 2014), including the infection zone which contain the cells becoming infected by rhizobia. A subsequent study, which transcriptionally profiled root hairs from seedlings undergoing infection (Breakspear et al., 2014), provides a unique opportunity to compare gene expression responses during infection of these two cell types. A disproportionate number of genes induced in root hairs of infected plants were expressed in the nodule apex. The majority of genes induced in root hairs in response to purified Nod factors were also expressed in the nodule apex (71% belonging to clusters 2–5), mirroring the analysis by Roux et al. (2014) who reported that 63.4% of genes induced by Nod factors in intact root samples in an earlier study (Czaja et al., 2012) belonged to clusters 1–4. This overlap suggests that a core set of genes required to support rhizobial infection in both epidermal and cortical (nodule) tissues are directly induced by Nod factor signaling.

### *ChOMT* Genes are Expressed Specifically in Infected Roots Hairs and in the Nodule Apex While Other Flavonoid Biosynthesis Genes are Expressed Throughout the Nodule

Expression of the *ChOMT* genes required for the production of the nod-gene inducing flavonoid methoxychalcone was high in infected root hairs and within the nodule was restricted to the infection zone, matching the pattern of expression of rhizobial Nod factor synthesis/export genes and the Nod factor receptors *Nod Factor Perception (NFP)* and *LysM receptor-like kinase (LYK3),* (**Figure 2**; Roux et al., 2014). This is consistent with the detection of flavonoids in root hairs undergoing infection (Hassan and Mathesius, 2012) and is consistent with evidence showing that Nod factor production by the rhizobia is required at all stages of infection thread development (Marie et al., 1992; Den Herder et al., 2007). While *ChOMT* expression was restricted to ZII, transcripts for genes required to make the methoxychalcone precursor isoliquiritigenin as well as rhizobial nod genes were also detected in the N-fixation zone (ZIII; **Figure 2**; Roux et al., 2014). The authors further showed using promoter-GUS analysis that the expression of the nod genes coincided with the relatively infrequent infection threads observed in ZIII. The significance of these sporadic infections which can also be observed in mature determinate nodules of *L. japonicus* (unpublished results) remains to be determined. Our analysis suggests that the nod gene inducer in the N-fixing zone of the nodule is unlikely to be methoxychalcone since *ChOMT* expression is tightly confined to the nodule apex. On the other hand isoliquiritigenin seems to fit the role as several genes required for its synthesis, including *CHR* and *Chalcone Isomerase*, have moderate levels of expression in ZIII. Another function recently highlighted for flavonoids is their role as antioxidants, having roles in stomatal closure and drought response (Nakabayashi et al., 2014; Watkins et al., 2014). It seems possible that these compounds could serve to help buffer against damage from ROS generated in cells undergoing infection. Two *Vestitone Reductase* genes needed for the production of medicarpin, one of which is expressed at infection sites in the epidermis (Breakspear et al., 2014), were also found to be expressed in ZI and the IZ (Supplementary Table S2). A potential role for medicarpin is selection against incompatible rhizobia and other opportunistic bacteria as previously discussed (Breakspear et al., 2014).

The expression of the nodD genes did not correspond with the Nod factor signaling domain that was clearly delineated by the expression of the Nod factor receptors, rhizobial nod factor biosynthesis genes, and the *ChOMTs*, with only one gene, nodD3, having about 30% of its expression in this domain and nodD1 and nodD2 being mainly expressed in ZIII. The lack of correspondence between nodD expression and the Nod factor signaling domain may reflect the fact that the nodD gene, at least in *Rhizobium leguminosarum*, is constitutively expressed and is not induced by flavonoids (Rossen et al., 1985). Furthermore, the NodD protein is present at relatively low levels in the rhizobia, suggesting that low constitutive expression is sufficient for its activity (Schlaman et al., 1991). While nodD expression is not induced by flavonoids, activation of nod genes by some NodD proteins is flavonoid-dependent; methoxychalcone strongly induces nod gene expression in rhizobia containing extra copies of nodD1 or nodD2 in *S. meliloti* (Hartwig et al., 1990). In contrast, NodD3 induction of nod gene expression is flavonoid-independent and requires the transcriptional regulator syrM (Mulligan and Long, 1989). Accordingly, in nodules syrM expression closely matches that of nodD3 Roux et al. (2014; Supplementary Table S2). A study which examined all three single nodD mutants showed a small reduction in nodulation in the nodD3 mutant only, but strongly reduced nodulation for all combinations of double mutants, suggesting all three isoforms are important in the interaction with *M. truncatula* (Smith and Long, 1998). Similar results were obtained with *M. sativa* (Honma and Ausubel, 1987). The available data suggest that nodD1 and nodD2 activation of nod gene expression in the infection zone occurs mainly through the action of methoxychalcone, but expression of these genes in ZIII may be mediated by another flavonoid, such as isoliquiritigenin, whilst NodD3 operates independently of flavonoids and is sufficient to sustain nodulation.

### *Rhizobial Induced Peroxidases* have Complementary Patterns of Expression in the Nodule

Also common to epidermal and cortical infection was the strong expression of a subset of *RIPs*. Type III peroxidases have been implicated in generation of apoplastic reactive oxygen species (Martinez et al., 1998; Bindschedler et al., 2006), with one important role being to promote cell wall hardening (Passardi et al., 2004). A corresponding role for these peroxidases has been proposed in the rigidification of the infection thread cell wall and matrix (Wisniewski et al., 2000). Expression of nine of the ten *RIPs* was strictly limited to Zones I and II of the nodule while another family member, *RIP9*, showed a complementary pattern, being very highly expressed (∼7 times higher than all other RIPs combined) in the IZ and ZIII. The expression of *RIP9* therefore coincides with the low pO_2_ in the proximal zones of the nodule; Roux et al. (2014) report that the leghemoglobin genes required for microaerobic conditions are strongly and abruptly upregulated in the IZ and remain highly expressed in ZIII. Indeed, low oxygen availability in these zones might explain the need for a highly expressed, separately regulated peroxidase isoform. While the link between type III peroxidases with rhizobial infection is well established (Cook et al., 1995; Ramu et al., 2002; this study) functional analysis is confounded by the presence of multiple family members with similar expression patterns. *RIP9* may therefore present an opportunity to use genetics to help study the role of these enzymes in nodulation.

Based on co-expression and co-regulation we identified candidate transcription factors involved in rhizobial infection. The group included *ERN1*, *ERN2*, *NSP2*, *NF-YA1*, and *NF-YC2*, all of which have been functionally implicated in rhizobial infection (Oldroyd and Long, 2003; Kaló et al., 2005; Combier et al., 2006, 2008; Heckmann et al., 2006; Middleton et al., 2007; Zanetti et al., 2010; Cerri et al., 2012; Soyano et al., 2013; Laporte et al., 2014). Two other genes encoding CCAAT-box subunits were also identified in the analysis, *NF-YA1*, a close homolog of *NF-YA1*, and *NF-YB7.* As these transcriptional regulators act in heterocomplexes having one of each A, B, and C subunits, it is tempting to speculate that these act together to control infection, with NF-YA1 and NF-YA2 acting interchangeably. Among the unstudied members of this group is a GRF zinc finger protein N20, which has expression that is highly nodulation-specific but with only weak aa sequence homology to other legumes. Also of interest is *ZPF6* which encodes a C2H2 transcription factor that is required for trichome development, and has been proposed as an integrator of GA and cytokinin signaling in this process (Zhou et al., 2013). Also found were the transcription factors cytokinin response regulator *RRA3*, an ERF (Medtr3g090760) with homology to *Arabidopsis Cytokinin Response Factor 4* (**Table 2**), and two genes encoding the GA biosynthetic enzymes, Ent-Kaurenoic Acid Oxidase 1 (KAO1) and Gibberellin 3-Oxidase 1 (GA3OX1; Supplementary Table S1), which were part of a larger set of GA and cytokinin related genes identified as upregulated after rhizobial inoculation (Breakspear et al., 2014; Liu et al., 2015). This indicates that the regulation of these hormones, along with auxin, is important during infection in both root hairs and within the nodule.

### Challenges and Future Directions

This study illustrates the power of single-cell type approaches to study the key mechanisms that underlie a biological process. Through the study of gene expression associated with infection thread formation in two different tissues, tissue-specific and background effects can be eliminated and core processes exposed. Improvements can of course still be made; Roux et al. (2014) estimated a 10% rate of contamination of ZII transcripts in the ZI sample, which argues the need for sampling smaller areas of tissue. Furthermore, it can be difficult to discern different cell types, for instance between meristematic cells and cells of the distal infection zone (Limpens et al., 2013). Similarly, while root hair isolation methods used by Breakspear et al. (2014) or Libault et al. (2010) are technically less challenging than laser-capture microdissection, only a small percentage of root hairs in the sample are undergoing infection. Ultimately, a single cell-based approach offers the greatest resolution, having the potential to sub-classify cells from the same tissue. Such an approach would avoid the averaging out of highly localized phenomena such as the production of superoxide reported in this study. Another limitation of the described approach is evident from the analysis of the transcriptional regulators. It is clear that infection thread development is comprised of numerous processes occurring in parallel sometimes within the same cells which cannot easily be separated even using single-cell approaches. This can be addressed in three ways. The first is to use a developmental time series, as employed in Breakspear et al. (2014), which allowed partial resolution of events occurring before and during infection. The second is the use of relevant sensory or chemical inputs that can perturb individual components of the system. For instance a subset of the genes may be inducible by treatment with ROS, allowing them to be partitioned away from the larger set of co-regulated genes. In this respect the ever-growing gene expression atlases available for medicago and other plants present a useful resource (Benedito et al., 2008; Hruz et al., 2008). The third and most powerful approach is the use of mutants. Careful comparison of specific mutants defective in one or a few processes will provide a clearer picture of the transcriptional network underlying rhizobial infection.

Our work compares two different types of data sets and identifies a core set of infection genes common to infection in both root hairs and nodules with specific attention to transcription factors that can serve as a starting point for future studies. In addition we show for the first time the expression pattern of two genes encoding *ChOMT* isoforms, an enzyme that plays a key role in the symbiosis that so far has only been studied at the biochemical level. We confirmed expression of these genes in the nodule infection zone, and have extended this knowledge by showing that these genes are specifically induced in infected root hairs, and that one gene is expressed in the nodule vascular bundle. We show using NBT staining that superoxide is being produced specifically in cells undergoing infection in root hairs and the nodule further demonstrating the tight link between ROS production and the infection process.

## Conflict of Interest Statement

The authors declare that the research was conducted in the absence of any commercial or financial relationships that could be construed as a potential conflict of interest.
